# Brainstem substructures and cognition in prodromal Alzheimer’s disease

**DOI:** 10.1007/s11682-021-00459-y

**Published:** 2021-03-01

**Authors:** Shubir Dutt, Yanrong Li, Mara Mather, Daniel A. Nation, for the Alzheimer’s Disease Neuroimaging Initiative

**Affiliations:** 1 Department of Psychology, University of Southern California, Los Angeles, CA USA; 2 Davis School of Gerontology, University of Southern California, Los Angeles, CA USA; 3 Institute for Memory Impairments and Neurological Disorders, University of California, Irvine, Irvine, CA USA; 4 Department of Psychological Science, University of California, Irvine, Irvine, CA USA

**Keywords:** Alzheimer’s disease, Brainstem, Cognition, Locus coeruleus, Magnetic resonance imaging

## Abstract

Neuropathological research suggests the tau pathology of Alzheimer’s disease may originate in brainstem nuclei, yet it remains unknown whether tau-mediated degeneration of brainstem nuclei influences cognitive impairment in prodromal Alzheimer’s disease. The present study examined cognitive domains impacted in prodromal Alzheimer’s disease and brainstem substructure volume in cognitively normal older adults (*n* = 814) and those with mild cognitive impairment (*n* = 542). Subsamples of cognitively normal (*n* = 112) and mild cognitive impairment (*n* = 202) also had cerebrospinal fluid Alzheimer’s disease biomarker characterization. Region-of-interest and voxel-level analyses related whole brainstem, midbrain, pons, and locus coeruleus volumes to cognition with multiple linear regression models corrected for age, sex, education, apolipoprotein-ε4 carrier status, and MRI magnet strength. Within mild cognitive impairment participants, smaller midbrain and locus coeruleus volumes were significantly related to poorer performance on tests of attention and executive function, and the relationship between locus coeruleus volume and executive abilities remained significant in the mild cognitive impairment subsample with biomarker-confirmed Alzheimer’s disease. A brainstem-masked voxel-wise regression further demonstrated an association between locus coeruleus volume and executive abilities. Brainstem volumes were not significantly related to memory processes. Study findings implicate midbrain and locus coeruleus volume in attention and executive deficits in mild cognitive impairment. Together with prior neuropathological studies, our data suggest a link between Alzheimer’s disease-related degeneration of brainstem nuclei and cognitive deficits in prodromal Alzheimer’s disease.

**Supplementary Information:**

The online version contains supplementary material available at 10.1007/s11682-021-00459-y.

## Introduction

Recent updated Braak staging of Alzheimer’s disease (AD) implicates the brainstem as the first site of tau-related pathology, with the locus coeruleus (LC) the first nucleus to demonstrate signs of pretangles (i.e., precursors to neurofibrillary tangle pathology) (Braak and Del Tredici 2015). Although the origin of tau seeding activity remains controversial, recent histopathological studies demonstrated the presence of tau cytoskeletal pathology in the LC prior to allocortical cytoskeletal changes (Heinsen and Grinberg 2018; Kaufman et al. 2018; Rüb et al. 2016; Stratmann et al. 2016). The LC is the noradrenergic epicenter of the brain and helps regulate autonomic and neurovascular function and modulate aspects of cognition. Human and animal studies reveal the LC-noradrenergic system modulates attentional shifts, executive function, cognitive control and memory processes (Aston-Jones and Cohen 2005; Mather 2020; Mather et al. 2016; Sara 2009). Recent efforts have highlighted the importance of characterizing LC integrity in aging and neurodegenerative disease (Mather 2020; Mather and Harley 2016), and neuroimaging studies have employed T1-weighted neuromelanin-sensitive scans to approximate LC structural integrity in vivo (Betts et al. 2019; Liu et al. 2017). Neuroimaging studies using these specialized scans have demonstrated associations between LC integrity and episodic memory encoding for stimuli of varying salience (Dahl et al. 2019; Hämmerer et al. 2018; Liu et al. 2020; Olivieri et al. 2019). However, to our knowledge no studies have evaluated associations between cognition and LC volume derived from standard structural T1-weighted scans.

Prior studies examining brainstem volumetrics with standard structural T1-weighted scans in AD populations found volume differences in rostral midbrain and pons regions in AD relative to cognitively normal (CN) individuals (Ji et al. 2020; Lee et al. 2015). Furthermore, we recently demonstrated volumetric differences specific to the midbrain and LC in the prodromal phase of AD, mild cognitive impairment (MCI), compared to CN individuals, and at an earlier preclinical stage in asymptomatic CN individuals who later received a diagnosis of AD dementia (Dutt et al. 2020). The methodology from this study adjusted for total brainstem volume and found overlap with prior LC masks, demonstrating that functionally-relevant LC volume estimates can be quantified from standard T1-weighted MRI scans. Thus, brainstem substructures, and the LC in particular, experience volumetric loss detectible on traditional MRI sequences during the early preclinical phase of AD pathophysiology. However, no studies have evaluated whether AD-related brainstem volume changes are associated with cognitive deficits. The present study investigated how neuropsychological deficits associated with brainstem substructure volume in prodromal AD, building upon our prior efforts to detail brainstem substructure volumes across the AD spectrum (Dutt et al. 2020). Based on the growing literature linking LC integrity with cognition, we hypothesized that smaller brainstem substructure volumes, and smaller LC volumes in particular, would be linked to worse performance on tests of attention, executive function and episodic memory encoding.

## Methods

### Study design

Data were obtained from the Alzheimer’s Disease Neuroimaging Initiative (ADNI) online database. The ADNI is a multisite natural history study that has collected clinical, biomarker, and neuropsychological data since 2003 to measure progression of normal aging, MCI, and AD. Detailed study information is available online (http://adni.loni.usc.edu/). 1356 participants with a baseline clinical diagnosis of CN or MCI and available neuropsychological and structural neuroimaging data were included from the ADNI1, ADNI GO, and ADNI2 cohorts. Participant data represented a subset of a larger study of brainstem volumetrics in preclinical and prodromal AD (Dutt et al. 2020). This study was conducted in accordance with the Helsinki Declaration and approved by all local Institutional Review Boards.

### Neuropsychological testing

Participants completed a standardized battery of neuropsychological tests at baseline. Trail Making Test parts A & B assessed attentional/executive abilities (visual attention & set-shifting, respectively). Rey Auditory Verbal Learning Test (RAVLT) delayed free recall and recognition assessed memory consolidation/retrieval abilities. RAVLT trial 1 performance assessed auditory attention and working memory, while RAVLT trials 1–5 total score indexed episodic memory encoding. Category fluency (Animals) tested both language (semantic retrieval) and executive abilities, while the Boston Naming Test (BNT) assessed language (confrontation naming) specific to lexical-semantic retrieval abilities.

### Cluster-derived diagnoses

We entered all participants clinically diagnosed as MCI at baseline into a cluster analysis to address previously described high rates of MCI misclassification (Bondi et al. 2014; Clark et al. 2013; Delano-Wood et al. 2009; Edmonds et al. 2015). First, participants diagnosed as CN by ADNI and who remained CN throughout enrollment were designated the normal reference group. Linear regression models predicted cognitive performance on six tests (Trails A, Trails B, RAVLT free recall, RAVLT recognition, Animals fluency, Boston Naming Test) from age and education within this normal reference group. Expected cognitive performance of MCI participants based on their age and education was calculated using the resulting regression coefficients from these models, and the expected scores were used along with the MCI participants’ observed performance to calculate age- and education-adjusted z-scores. Finally, z-scores were entered into a hierarchical cluster analysis using Ward’s method and a forced 4-cluster solution. An emergent cluster-derived CN group was combined with the ADNI-diagnosed CN group to form the CN group (*n* = 814), while the remaining three MCI sub-groups (amnestic, dysnomic, and dysexecutive) formed the MCI group (*n* = 542).

### Neuroimaging acquisition & analyses

T1-weighted structural images were collected from all ADNI participants using either a 3D-MPRAGE or 3D IR-SPGR sequence. Sequence parameters are available online (http://adni.loni.usc.edu/methods/documents/mri-protocols/). MRI scans from 1.5 T and 3 T magnetic field strengths were combined for analyses, an approach previously shown to be feasible in voxel-based analyses of the ADNI dataset (Dutt et al. 2020; Jack et al. 2015; Marchewka et al. 2014). Images were downloaded from the ADNI-LONI database, checked for image quality, and manually reoriented in SPM12 within MATLAB (http://www.fil.ion.ucl.ac.uk/spm/). Images were processed using the voxel-based morphometry (VBM) pipeline via segmentation into tissue classes, creation of and alignment to a study-specific DARTEL template, spatial normalization, modulation, and 8 mm smoothing (Ashburner and Friston 2000). Region-of-interest (ROI) masks for midbrain, pons, and whole brainstem were derived from previously published atlases (Iglesias et al. 2015; Mazziotta et al. 2001). We used a pre-existing LC ROI mask that averaged peak voxel coordinates from studies that localized the LC on functional MRI and neuromelanin-sensitive T1-weighted scans (https://rcweb.dartmouth.edu/CANlab/brainstemwiki/doku.php/lc.html) (Astafiev et al. 2010; Keren et al. 2009). To adjust for whole brain volume and facilitate comparisons, we divided ROI volumes by total intracranial volume and multiplied them by 10^3^ (midbrain, pons, whole brainstem) or 10^4^ (LC) (Whitwell et al. 2001).

### CSF biomarkers

MCI participants who were both amyloid-β (Aβ) and phosphorylated tau (pTau) positive based on pre-established cutoffs (Hansson et al. 2018) comprised the MCI due to AD group (MCI_Aβ + pTau+_, *n* = 202). Aβ-positive and pTau-positive CN participants comprised the preclinical AD group (CN_Aβ + pTau+_, *n* = 112). For detailed information on CSF biomarker quantification, see Supplemental Methods.

### Statistical analyses

For all ROI volumes and cognitive measures, Pearson correlations were first examined to confirm the presence or absence of zero-order relationships (Keith 2014; Kraha et al. 2012), followed by multiple linear regression models with TIV-adjusted brainstem ROI volume as independent variable, neuropsychological test as dependent variable, and age, sex, education, apolipoprotein-ε4 (*APOE*-ε4) carrier status, and MRI magnet strength as covariates. In order to demonstrate that our substructural findings were independent of total brainstem volume changes, we repeated analyses with an additional covariate for total brainstem volume. False discovery rate (FDR) correction via the Benjamini-Hochberg procedure (Glickman et al. 2014) was controlled at 0.10 to address multiple comparisons, similar to prior AD studies (Readhead et al. 2018; Yew and Nation 2017). Further information regarding statistical analyses is available in Supplemental Methods.

For all significant multiple regressions, we conducted exploratory voxel-wise regression analyses in SPM12 with neuropsychological test of interest as independent variable and segmented white matter map as dependent variable, consistent with prior studies (Dutt et al. 2016, 2020; Nigro et al. 2014). An explicit mask of the midbrain and pons constrained analyses to rostral brainstem regions, and age, sex, education, *APOE*-ε4 carrier status, MRI magnet strength, and total intracranial volume were included as covariates. Voxel-wise analyses were repeated with an additional covariate for pons volume to determine regional specificity. Results were examined at family-wise error (FWE)-corrected *p* < 0.05 and uncorrected *p* < 0.05.

## Results

### Demographic, clinical, and cognitive variables

Descriptive statistics for demographic, cognitive, and neuroimaging variables are displayed in Table 1.

**Table 1:** Descriptive statistics for demographic, cognitive, and neuroimaging variables

	Total Sample		Prodromal AD Subsets	
	CN	MCI	CN_Aβ + pTau+_	MCI_Aβ + pTau+_
Demographics				
*n*	814	542	112	202
Age	73.49 (6.76)	73.54 (7.35)	74.75 (6.18)	73.61 (7.13)
Sex (M/F)	417/397	332/210	59/53	112/90
Education	16.29 (2.65)	15.85 (2.92)	15.90 (2.66)	15.98 (2.86)
* APOE*-ε4 (0/1/2 ε4)	536/246/32	249/221/72	39/61/12	57/104/41
MRI Scanner (1.5 T/3 T)	304/510	305/237	34/78	91/111
Cognitive Testing				
Trails A	−1.53 (0.14)	−1.60 (0.17)	−1.56 (0.14)	−1.63 (0.17)
Trails B	−1.91 (0.17)	−2.06 (0.23)	−1.97 (0.19)	−2.08 (0.21)
RAVLT Trial 1	5.23 (1.78)	4.14 (1.41)	4.81 (1.62)	4.01 (1.38)
RAVLT Encoding	43.47 (10.42)	30.10 (8.14)	39.88 (9.56)	28.71 (7.39)
RAVLT Recall	7.26 (3.88)	2.16 (2.62)	6.11 (3.39)	1.55 (2.23)
RAVLT Recognition	13.05 (2.20)	8.93 (3.21)	12.97 (2.09)	8.58 (3.07)
Animals Fluency	20.13 (5.26)	15.73 (4.72)	19.22 (4.60)	15.46 (4.59)
BNT	−0.37 (0.28)	−0.65 (0.34)	−0.42 (0.27)	−0.67 (0.32)
Neuroimaging				
TIV	1499.92 (146.93)	1518.99 (159.50)	1488.19 (146.29)	1505.68 (166.34)
LC	1.22 (0.12)	1.20 (0.12)	1.24 (0.13)	1.21 (0.11)
Midbrain	3.88 (0.31)	3.82 (0.31)	3.92 (0.30)	3.85 (0.30)
Pons	7.70 (0.75)	7.60 (0.75)	7.81 (0.81)	7.67 (0.73)
Brainstem	13.32 (1.18)	13.14 (1.20)	13.50 (1.23)	13.26 (1.17)

Means (SD) are reported for continuous variables unless otherwise noted. Biomarker-positive groups are subsets of respective diagnostic groups. ROI volumes (LC, midbrain, pons, brainstem) were normalized via division by TIV. Scores for Trails A, Trails B, and BNT were log-transformed and reflected

*Aβ*, amyloid-β; *APOE*-ε4, apolipoprotein ε4; *BNT*, Boston Naming Test; *CN*, cognitively normal; *LC*, locus coeruleus; *MCI*, mild cognitive impairment; *pTau*, phosphorylated tau; *RAVLT*, Rey Auditory Verbal Learning Test; *ROI*, region of interest; *TIV*, total intracranial volume

### Memory

Multiple linear regression models predicting memory performance (RAVLT trials 1–5, delayed recall, and recognition) from ROI volumes were not significant within CN, MCI, CN_Aβ + pTau+_, or MCI_Aβ + pTau+_.

### Attention and executive function measures

**Fig. 1:**
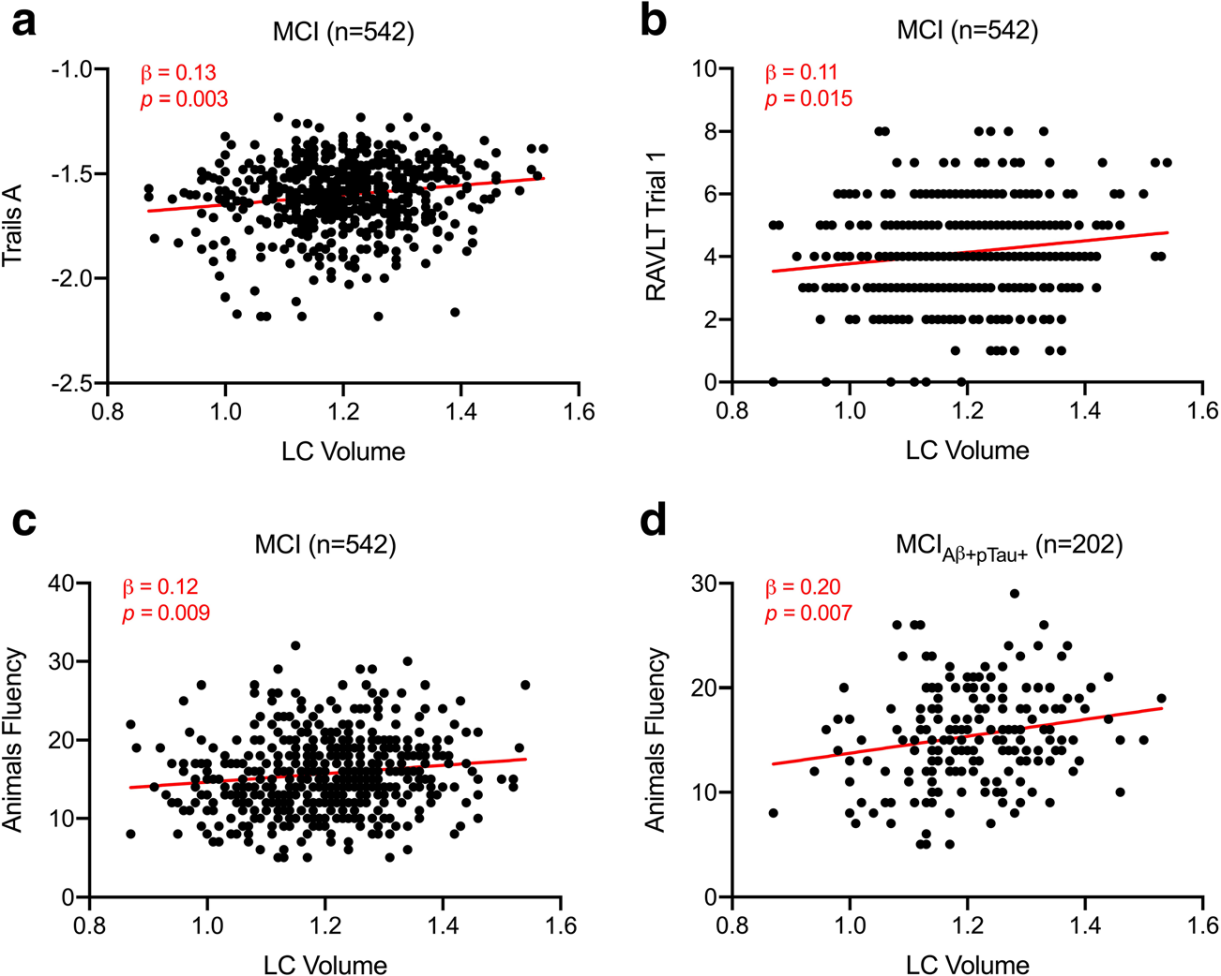
Regression analyses predicting cognition from locus coeruleus volume. Scatter plots and regression lines showing associations between TIV-normalized LC volume and (**a**) Trails A performance, (**b**) RAVLT trial 1 performance, and (**c**) category fluency performance in the MCI group (*n* = 542), and (**d**) between LC volume and category fluency performance in the MCI_Aβ + pTau+_ group (*n* = 202). Plotted data are unadjusted values, and red text indicates β and *p* value corresponding to multiple linear regression models with ROI volume as independent variable, cognitive test as dependent variable, and age, sex, education, *APOE*-ε4 carrier status, and MRI magnet strength as covariates. Abbreviations: Aβ = amyloid-β, *APOE*-ε4 = apolipoprotein ε4, LC = locus coeruleus, MCI = mild cognitive impairment, pTau = phosphorylated tau, RAVLT = Rey Auditory Verbal Learning Test, ROI = region of interest, TIV = total intracranial volume.

Within the overall MCI group, multiple linear regression models indicated smaller LC volume predicted worse performance on Trails A (β = 0.13, *p* = 0.003; Fig. 1a), RAVLT trial 1 (β = 0.11, *p* = 0.015; Fig. 1b), and Animals fluency (β = 0.12, *p* = 0.009; Fig. 1c). When including an additional covariate for whole brainstem volume, the relationship between LC volume and Animals fluency (β = 0.29, *p* = 0.008) remained significant. When constraining analyses to AD biomarker-positive MCI_Aβ + pTau+_ participants, smaller LC volume predicted worse performance on Animals fluency (β = 0.20, *p* = 0.007; Fig. 1d), and this finding remained significant with an additional covariate for whole brainstem volume (β = 0.48, *p* = 0.007).

**Fig. 2:**
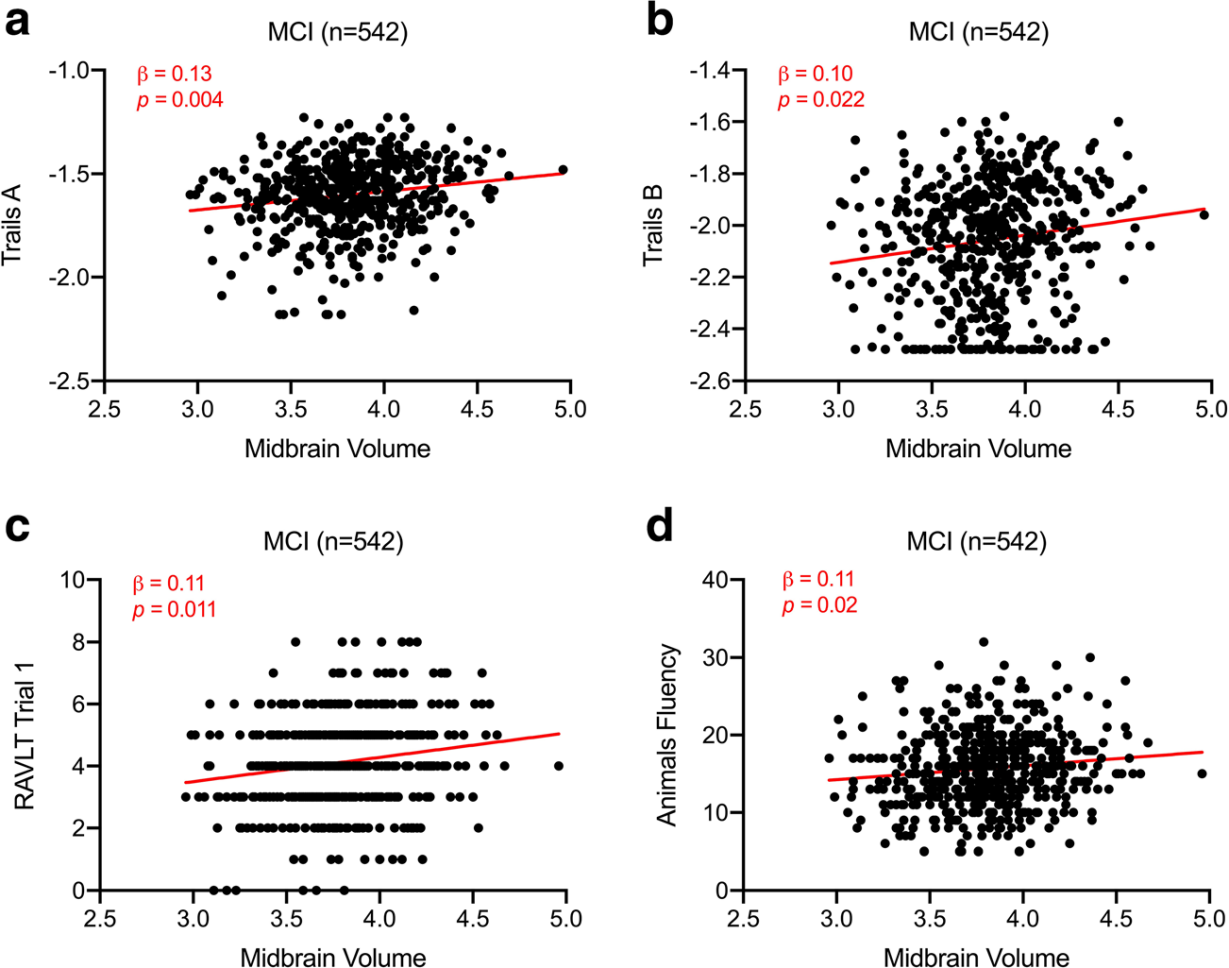
Regression analyses predicting cognition from midbrain volume. Scatter plots and regression lines showing associations between TIV-normalized midbrain volume and (**a**) Trails A performance, (**b**) Trails B performance, (**c**) RAVLT trial 1 performance, and (**d**) category fluency performance in the MCI (*n* = 542) group. Plotted data are unadjusted values, and red text indicates β and *p* value corresponding to multiple linear regression models with ROI volume as independent variable, cognitive test as dependent variable, and age, sex, education, *APOE*-ε4 carrier status, and MRI magnet strength as covariates. Abbreviations: Aβ = amyloid-β, *APOE*-ε4 = apolipoprotein ε4, LC = locus coeruleus, MCI = mild cognitive impairment, pTau = phosphorylated tau, RAVLT = Rey Auditory Verbal Learning Test, ROI = region of interest, TIV = total intracranial volume

Within the overall MCI group, smaller midbrain volume predicted worse performance on Trails A (β = 0.13, *p* = 0.004; Fig. 2a), Trails B (β = 0.10, *p* = 0.022; Fig. 2b), RAVLT trial 1 (β = 0.11, *p* = 0.011; Fig. 2c), and Animals fluency (β = 0.11, *p* = 0.02; Fig. 2d), while smaller whole brainstem volume (β = 0.10, *p* = 0.02) and smaller pons volume (β = 0.09, *p* = 0.031) predicted worse performance on Trails A. When correcting for whole brainstem volume, smaller midbrain volume predicted worse performance on Trails B (β = 0.28, *p* = 0.016) and RAVLT trial 1 (β = 0.26, *p* = 0.026). Within AD biomarker-positive MCI_Aβ + pTau+_ participants, midbrain, pons, or whole brainstem volumes were not associated with neuropsychological testing. Regression models predicting attention and executive function performance from ROI volumes were not significant within the CN or CN_Aβ + pTau+_ groups.

**Fig. 3:**
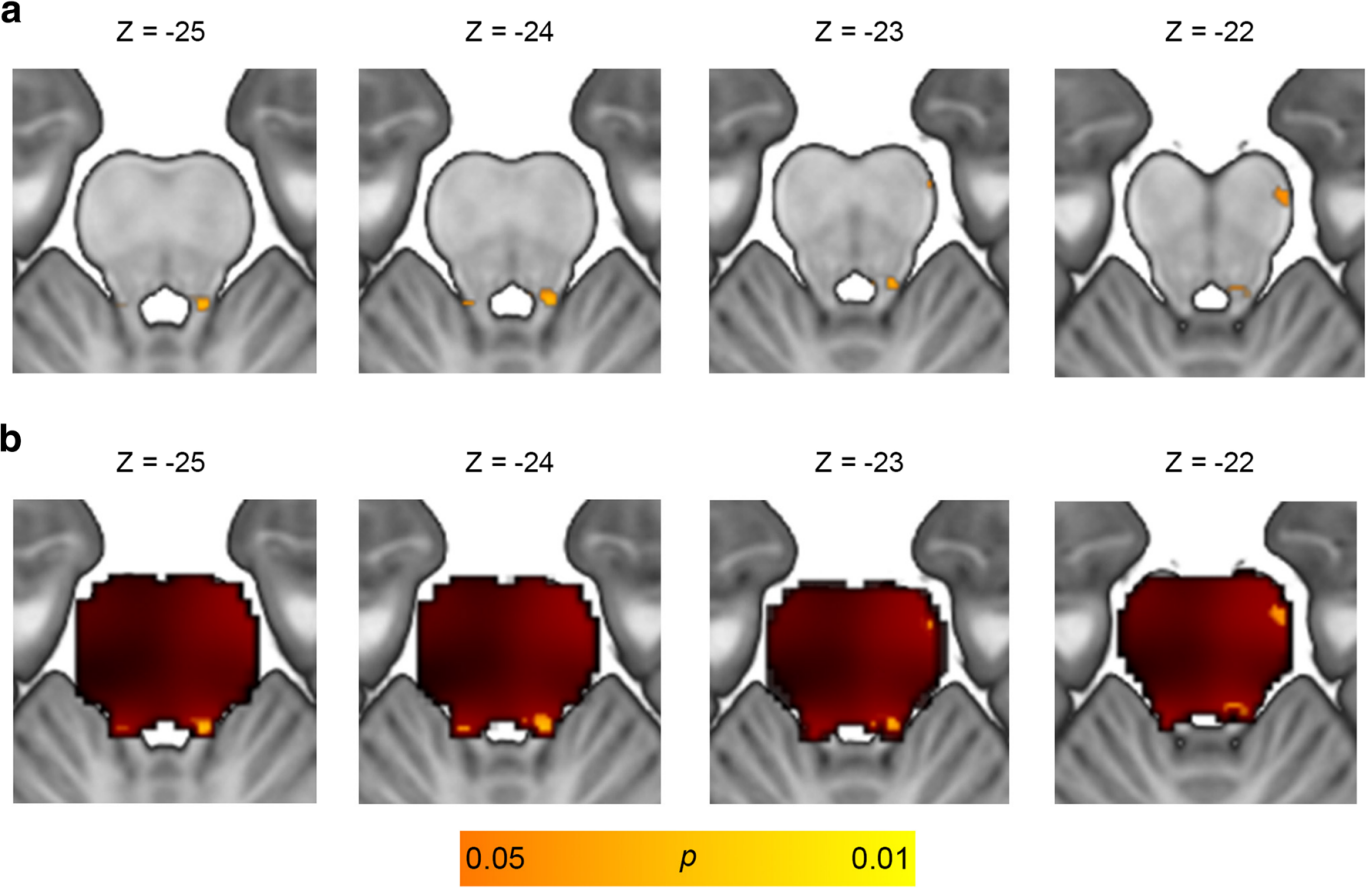
Voxel-wise correlation between category fluency and locus coeruleus volume. Results of voxel-wise multiple regression correlating brain volume with category fluency performance in the MCI (*n* = 542) group with covariates for total intracranial volume, age, sex, education, *APOE*-ε4 carrier status, and MRI magnet strength. (**a**) Significant clusters emerged overlapping the bilateral locus coeruleus and right lateral midbrain at an uncorrected height threshold of *p* < 0.05. (**b**) Significant clusters at *p* < 0.05 (orange) overlaid on an unthresholded statistical map (red). Explicit mask comprising the midbrain and pons was applied to limit search volume to rostral brainstem structures. Images are shown in neurological orientation. Text indicates MNI coordinates of corresponding axial slices. Abbreviations: *APOE*-ε4 = apolipoprotein ε4, MCI = mild cognitive impairment, MNI = Montreal Neurological Institute

Brainstem-masked voxel-wise regressions relating brain volume to neuropsychological tests within the overall MCI group were not significant at FWE-corrected *p* < 0.05. At a less stringent threshold of uncorrected *p* < 0.05, worse Animals fluency correlated with smaller volume of clusters overlapping the bilateral LC and right anterolateral midbrain (Fig. 3; Table 2), and a similar cluster emerged when including an additional covariate for total pons volume (Supp Fig. 1, Supp Table 1).

**Table 2:** MNI coordinates from voxel-wise correlation between category fluency and locus coeruleus volume

**A**							
Set-level	Cluster-level		Peak-level				
*p*	*p*_uncorr_	k_E_	*p*_uncorr_	T	x	y	z
0.032	0.983	19	0.023	1.99	8	−40	−24
	0.994	4	0.036	1.79	−8	−40	−22
	0.987	13	0.043	1.70	14	−21	−21
**B**							
		MNI x-range		MNI y-range		MNI z-range	
Voxel-wise correlation with Animals fluency		8 to −9		−38 to −41		−21 to −27	
MCI < CN (Dutt et al. 2020)		8 to −8		−39 to −42		−21 to −28	
AD < CN (Dutt et al. 2020)		9 to −6		−33 to −41		−17 to −26	
Converters < Non-Converters (Dutt et al. 2020)		8 to −8		−39 to −41		−21 to −26	
LC mask (Keren et al. 2009)		9 to −9		−36 to −39		−18 to −33	
LC mask (Betts et al. 2017)		9 to −9		−36 to −43		−15.5 to −37.5	
LC mask (Dahl et al. 2019)		8 to −10		−29 to −42		−18 to −38	
NC vs. AD peak coordinates (Ji et al. 2020)		−6, −9		−36, −36		−24, −29	

(**A**) Coordinates from voxel-wise multiple regression in MCI (*n* = 542) group regressing category fluency onto brain volume with an explicit mask comprising the midbrain + pons and covariates for total intracranial volume, age, sex, education, *APOE-*ε4 carrier status, and MRI magnet strength. (**B**) MNI coordinate range for significant clusters from present study and from prior brainstem VBM studies and established locus coeruleus masks

*AD*, Alzheimer’s disease; *CN*, cognitively normal; *k*_*E*_, cluster size; *LC*, locus coeruleus; *MCI*, mild cognitive impairment; *NC*, normal controls; *MNI*, Montreal Neurological Institute; *uncorr*, uncorrected

### Language

Multiple regression models predicting BNT performance from ROI volumes were not significant within participant subgroups (CN, MCI, CN_Aβ + pTau+_, MCI_Aβ + pTau+_).

## Discussion

The present study found that MCI patients with smaller midbrain and LC volumes performed worse on tests of visual attention (Trails A), verbal attention (RAVLT trial 1), executive function (Trails B), and category fluency (Animals), suggesting brainstem substructural volumes may be related to underlying attention, processing speed, and executive abilities. In MCI patients with biomarker-confirmed AD, the relationship between LC volume and Animals fluency remained significant in the presence of prodromal AD pathology. Whole brainstem, midbrain, pons and LC volumes were not associated with episodic memory (RAVLT encoding, delayed recall, and recognition) or a confrontation naming test of language ability (BNT), highlighting the specific association between brainstem substructure volumes and measures of attention, processing speed, and executive function. This is the first study to report associations between cognition and brainstem substructure volumes in MCI populations. We provided preliminary evidence that well-documented relationships between the LC noradrenergic system and attention (Aston-Jones et al. 1999; Mather et al. 2020; Sara 2009) are detectible when examining LC volume in the prodromal phase of AD.

The critical MCI phase preceding AD dementia may be a window when neural and cognitive reserve in brainstem regions are integral to maintaining optimal cognitive function. Within this prodromal period, we found that individuals with smaller midbrain and LC volumes performed worse on tasks of executive function and visual and verbal attention. This echoes the neuropathology literature demonstrating that individuals with greater pathological burden (i.e., greater subcortical tau deposition) exhibit diminished volumes of nuclei known to contain the first signs of AD-related pathology and perform worse on corresponding cognitive tests (Braak and Del Tredici 2015; Grudzien et al. 2007). Alternatively, our findings could reflect that greater premorbid LC volume supports better performance on attentional tasks. This supports a previously theorized buffering role of the LC, due to its high lifetime noradrenergic turnover and neuronal density, in protecting against the detrimental effects of accumulating AD-related pathology (Clewett et al. 2015; Mather and Harley 2016; Robertson 2013). Although the exact role of brainstem degeneration in cognitive dysfunction is not well-understood, degeneration of the LC appears to be related to cognitive function in normal aging (Dahl et al. 2019; Langley et al. 2020) and correlates with cognitive abilities and pathological protein accumulation in animal models of AD (Chalermpalanupap et al. 2017; James et al. 2020; Kelberman et al. 2020). Of note, we found attenuated brain-behavior relationships in the biomarker-confirmed MCI due to AD group compared to the overall MCI group, likely due to the smaller sample size. Interestingly, we did not observe relationships between brainstem structure and cognition in the CN group, despite observable first signs of tau pathology in postmortem adult cognitively normal samples (Braak and Del Tredici 2015). We previously demonstrated that LC structural abnormalities are observable using MRI with cognitively normal participants (Dutt et al. 2020); however, the current findings suggest these individuals do not yet exhibit cognitive decline that correlates with brainstem structure. Future studies will be necessary to clarify whether LC function, as opposed to structure, in the early preclinical AD phase better correlates with cognition.

The category fluency task was the cognitive test most strongly associated with midbrain and LC volumes in MCI and biomarker-confirmed MCI due to AD. The category fluency task, though often broadly categorized under the domain of language processing, also requires executive abilities subserved by frontal-subcortical systems, including monitoring, shifting, and inhibition (Shao et al. 2014). Furthermore, the category fluency task is similar to other tests from the present study (e.g., Trails A & B) because it represents a timed test requiring adequate attention and processing speed to complete successfully (Auriacombe et al. 2001; Baddeley and Della Sala 1996). Subcortical dementias experience specific impairments in attention, executive function, and processing speed (Cummings 1986; Salmon and Filoteo 2007), and our findings may similarly reflect subcortical contributions to cognitive impairment in prodromal AD.

The present study did not find relationships between brainstem volumes and episodic verbal memory encoding, which contrasts with associations observed in studies of LC signal intensity and memory encoding performance during verbal learning and immediate recall tasks in older adults and AD populations (Dahl et al. 2019; Olivieri et al. 2019). Memory performance on the immediate recall trial and across the encoding trials is linked to an individual’s ability to engage attention during the presentation of stimuli and store items in working memory (Buckner et al. 2000), and our study findings suggest a role of brainstem volume in attention and working memory. Interestingly, relationships between brainstem volumes and measures of episodic verbal memory abilities linked to integrity of medial temporal and hippocampal structures (Squire and Zola-Morgan 1991), were not observed. Our approach did not include hippocampal and medial temporal structures, as these areas are well-studied and known to experience profound atrophy in AD neurodegenerative processes (Jack et al. 1998; Mori et al. 1997). Our study was not designed to determine if brainstem substructures are better predictors of cognition than medial temporal and hippocampal regions, but rather to independently assess relationships between brainstem substructure volumes and cognition. Our findings complement a growing body of evidence supporting the role of LC structural integrity (as measured by neuromelanin-sensitive T1-weighted imaging) and functional activity (as measured by fMRI) in diverse memory processes when the stimuli involved are particularly salient or emotionally charged (Clewett et al. 2018; Hämmerer et al. 2018; Jacobs et al. 2020; Liu et al. 2020). The relative neutrality of word stimuli in the present study may partially explain why no relationships between brainstem volumes and recall or recognition were observed, yet a recent diffusion-weighted imaging study found an association between LC microstructure and RAVLT delayed recall performance in healthy older adults (Langley et al. 2020). More multimodal neuroimaging work is needed in MCI populations to disentangle the specific associations between brainstem substructures and memory for stimuli of varying emotional arousal.

A study limitation is the use of segmented structural T1 images to estimate volumes of deep brainstem nuclei, which inherently lack information regarding the boundaries of structures such as the LC. Although prior studies have demonstrated an ability to detect structural brainstem differences between disease groups with a similar method (Dutt et al. 2020), our approach should be further validated in cohorts with MRI sequences specialized for assessment of LC structure (Betts et al. 2019). Another limitation is the racially homogeneous and highly educated nature of the ADNI cohort, which limits the generalizability of our findings. Future studies should examine diverse populations. Given the cross-sectional study design, directionality of brainstem-cognition relationships cannot be determined. Other limitations include the overlaid ROI approach to volume extraction as opposed to individual structural segmentation and the high variability in individual subject history and instrumentation between sites, all of which should be addressed in follow-up studies.

## Conclusions

The present study examined relationships between brainstem volumes and cognition by quantifying VBM-estimated brainstem substructure and LC volumes from structural MRI images in individuals with normal cognition, biomarker-confirmed preclinical AD, neuropsychologically-confirmed MCI, and biomarker-confirmed MCI due to AD. Midbrain and LC volumes were associated with measures of attention, processing speed, and executive function, but not with episodic memory performance or confrontation naming. A growing number of studies have implicated subcortical brainstem structures as the earliest sites of AD-related tau pathology, and MRI-measured volumes of these regions appear to correlate strongest with tasks that require greater executive control and attention in the MCI phase preceding the later onset of dementia.

## Supplementary Information

ESM 1 (DOC 142 kb)

## Data Availability

All data used in the present study are publicly available via the ADNI website (http://adni.loni.usc.edu/) and the ADNI-LONI Image & Data Archive (https://ida.loni.usc.edu/login.jsp).
