# Supplementary material for: Brainstem substructures and cognition in prodromal Alzheimer’s disease
Source: Brain Imaging Behav. 2021 Mar 1;15(5):2572–82. doi: 10.1007/s11682-021-00459-y (PMC8500899; doi:10.1007/s11682-021-00459-y)
Supplement: Supplementary file 1 — (DOC 142 kb) [file 11682_2021_459_MOESM1_ESM.doc]

**Supp. Fig. 1. Voxel-wise correlation between category fluency and locus coeruleus volume corrected for pons volume**

**
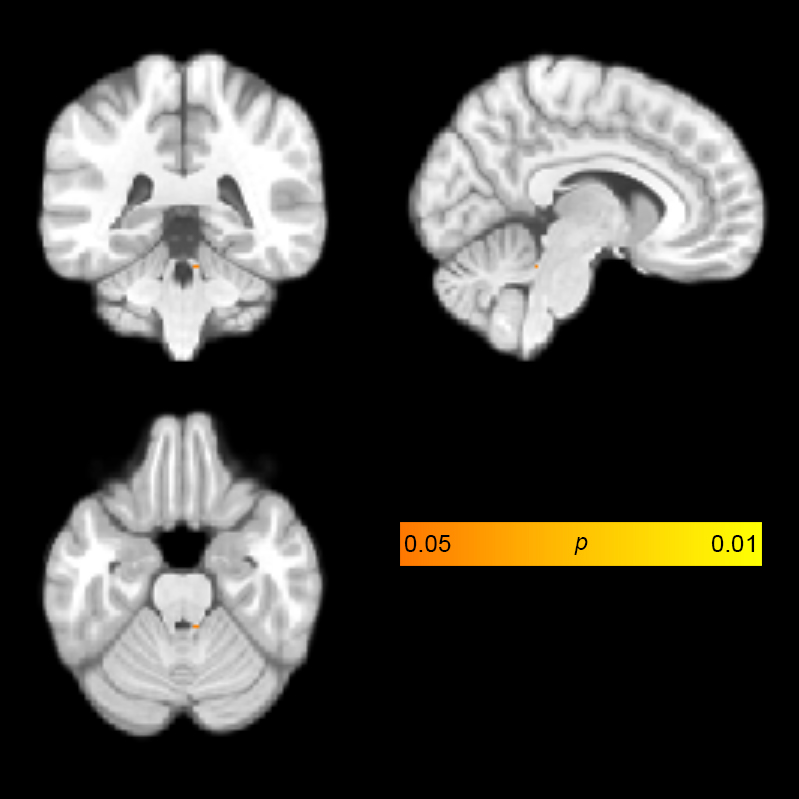
**

Results of voxel-wise multiple regression correlating brain volume with category fluency performance in the MCI (n = 542) group with covariates for age, sex, education, *APOE*-ε4 carrier status, MRI magnet strength, and total intracranial volume. Significant cluster emerged overlapping the right locus coeruleus at an uncorrected height threshold of *p* < 0.05. Explicit mask comprising the midbrain and pons was applied to limit search volume to rostral brainstem structures. Image is shown in neurological orientation.

**Supp. Table 1. MNI coordinates table for pons-corrected VBM analysis**

| Cluster-level | | Peak-level | |  |  |  |
| --- | --- | --- | --- | --- | --- | --- |
| *p*_uncorr_ | k_E_ | *p*_uncorr_ | T | x | y | z |
| 0.984 | 2 | 0.048 | 1.66 | 8 | -40 | -24 |

Coordinates represent finding from voxel-wise multiple regression in MCI (n = 542) group regressing category fluency onto brain volume with an explicit mask comprising the midbrain + pons and covariates for total intracranial volume, age, sex, education, *APOE-*ε4 carrier status, and MRI magnet strength. Abbreviations: k_E_ = cluster size, MCI = mild cognitive impairment, MNI = Montreal Neurological Institute, uncorr = uncorrected

**Supplemental Methods**

**CSF Biomarkers**

Levels of CSF amyloid-β (Aβ) 1-42 and phosphorylated tau (pTau) were quantified in aliquots using the automated Roche Elecsys β-amyloid (1-42) CSF and Elecsys phosphotau (181P) CSF electrochemiluminescene immunoassays at the UPenn Biomarker Research Laboratory; detailed information is available online (<http://adni.loni.usc.edu/methods/>). Participants were categorized as Aβ-positive and pTau-positive based on pre-established cutoffs of 980 pg/mL and 21.8 pg/mL, respectively (Hansson et al. 2018).

**Statistical Analyses**

Prior to analyses, distributions of continuous variables were checked for normality via skewness and kurtosis. The Trails A, Trails B, and BNT variables had highly skewed distributions and were corrected with log-transformation. Scores for Trails A and Trails B were reflected to ensure consistent directionality across all neuropsychological tests, with higher scores indicating better performance. BNT scores were reflected prior to log-transformation to avoid undefined values. Outliers ± 3 standard deviations from the mean were identified and analyses were run with and without these datapoints; none of the reported results were affected by the removal of outliers, thus all datapoints were included.
